# Designing a Facebook Interface for Senior Users

**DOI:** 10.1155/2014/741567

**Published:** 2014-02-02

**Authors:** Gonçalo Gomes, Carlos Duarte, José Coelho, Eduardo Matos

**Affiliations:** LaSIGE, University of Lisbon, Campo Grande, 1749-016 Lisboa, Portugal

## Abstract

The adoption of social networks by older adults has increased in recent years. However, many still cannot make use of social networks as these are simply not adapted to them. Through a series of direct observations, interviews, and focus groups, we identified recommendations for the design of social networks targeting seniors. Based on these, we developed a prototype for tablet devices, supporting sharing and viewing Facebook content. We then conducted a user study comparing our prototype with Facebook's native mobile application. We have found that Facebook's native application does not meet senior users concerns, like privacy and family focus, while our prototype, designed in accordance with the collected recommendations, supported relevant use cases in a usable and accessible manner.

## 1. Introduction

Increasing longevity is shifting the age distribution of the populations of industrialized countries toward older age groups. Some countries—such as Australia, Canada, France, Germany, Japan, New Zealand, the United Kingdom, and the United States—are experiencing the effects of population ageing on several services [1, 2]. In these countries, the ageing process is usually related with retirement, which in turn brings solitude and isolation, also resulting in several health problems [3]. Past works have shown that older adults are frequent users of technology, which is being designed to support an active life [4] in an attempt to mitigate those problems. Recent studies also show that the usage of platforms like Facebook, by allowing frequent, light and collective discussions with close family [5], increases well-being and life satisfaction [3], and reduces isolation [6]. For these reasons, the adoption of social networks by older adults has risen considerably in the recent years [7]. However, many still cannot make use of social networks as these are simply not designed for them [3].

Social Networks Services (SNS) are a particular set of social media supportive platforms that focus on providing viable ways of connecting users with each other [8]. The most widely used SNS, such as Facebook or Google+, have been designed for younger and average aged users, which results in interfaces that bring interaction challenges to users aged 65 and above [3]. One could consider designing a social network platform that would account for this design issue, thus trying to achieve the best interaction experience among the senior population. Still, this approach reveals two major limitations: (1) the difficulty of overcoming the challenge of attracting the masses to a newly created community service and (2) the lack of acceptance from nonsenior users of a service that would have been designed for an older age group. Within that nonsenior users group, the older adult's family is a particularly relevant set. As shown in [9], family is a fundamental factor when considering the senior population. Thus, the problem of connecting the older adult with their family would still remain.

These barriers must be broken by designing solutions which consider specific age related conditions from the start, enabling seniors to take advantage of new technologies and services that can help improve their quality of life, while using the same services that other age groups, and particularly their family, use. Considering this, in this paper we present a prototype built considering a set of design recommendations derived from direct inquiries of senior users of Facebook. Additionally, the paper presents a user study comparing the developed prototype with Facebook's native Android application, with a focus on the factors that are more relevant for senior users.

## 2. Related Work

### 2.1. Social Isolation

The ageing process is a considerable issue when focusing on the users' well-being, since one factor influences the other, as stated in [3]. In 2002, the World Health Organization adopted the expression “Active Ageing” [10] such that they could express the concept of ageing relative to the concepts of social participation and safety, and with the aim of improving the quality of life of the elderly [11]. A common factor among the elderly population is social isolation. Studies have revealed that social isolation is typically linked with poor health outcomes. For instance, David et al. [12] stated that it can even be a prominent feature of having chronic pain. Moreover, there are strong indicators that show that both frequency and perceived quality of contact affect the health outcomes of isolated older adults [13]. This statement is also supported by studies which revealed that chronic pain patients who perceived they had family support reported less pain intensity, more satisfactory social activity, and fewer instances of pain interfering with daily living than those who perceived their family support was somehow inadequate [14]. Such observations suggest that there is a preponderant role for communications technology to play in better supporting senior users.

Older adults are a very particular set of users within the population and possess several very specific characteristics that must be taken into consideration. Considering this range of population and their knowledge about technology, there is a requirement for the design of adaptive applications and thus, technology designers must understand relevant prior knowledge in a target user population to facilitate adoption and effective use. O'Brien et al. [15] made a study in which they systematically collected information about technologies used over a specific period by older adults with either low or high technological experience. Their results have suggested that a good design is still a crucial concept for everyday technology interaction, and that users expect information on everyday technology to direct them to helpful support. Thus, even though prior knowledge about some of everyday technology is a crucial concept for the definition of interaction expertise, it is still not the major concern, for it is also necessary to focus on design aspects that still affect users when interacting with some interfaces [4]. Typically, the most common interfaces with which seniors are used to interact with are the ones belonging to social networking sites, which already represent a very considerable number in terms of usage in the internet [8, 16].

### 2.2. Social Networks

Social networking sites have the potential to be the main equalizer when it comes to providing social communication between individuals. In the early days of the internet, Human-Computer Interaction (HCI) researchers had the tendency to treat both services and users monolithically [8], by assuming that internet usage had the same effect on most users. As an example, several early studies examined the association between overall time online, social capital, and loneliness [17]. As internet services have grown richer, researchers began to ask whether different types of internet usage—such as communicating with family—had different effects on those varying demographics [8, 18]. SNS are designed to connect people with friends, family, and other strong ties that might exist. Therefore, they have the required persuasive power in order to influence its users' psychological well-being that often flows from social capital [8]. Moreover, a successful SNS is one that provides a good support for communication between users and group-based interaction, as well as allowing activity-based behaviours for its users. Taking this into account, researchers are, nowadays, beginning to recognize that not all SNS use is equally “social” [19]. Literature references indicate that people derive benefits from their interpersonal relationships and groups they belong to, which can range from improved health to access to expertise and financial resources [8]. All these observations reveal an increasing need for interfaces that support simple interactions and improved user experience, especially for senior users.

### 2.3. Touch Interfaces

Touch interaction is widely used and it is spread among different devices, applications, and contexts. We are already using touchscreen functionalities on a daily basis without even noticing, mainly due to its enormous success on mobile devices, which in turn are indeed increasingly replacing keypad-based applications. This is justified by the fact that this type of interfaces provides the ability to directly touch and manipulate data on the screen of the interface without intermediate devices, thus providing a more natural and engaging experience for the user. Additionally, studies reveal that touchscreens offer high flexibility, which makes it possible to display several different interfaces on the same surface or even to adapt to the users' needs and/or preferences [20]. Despite this, and since many seniors were not reliant on computing machinery during their younger and working years, these individuals have become somewhat disconnected from some of the online activities of today's information society, as stated in [21]. Clearly there is a need for the development of more attractive and user-friendly interfaces in general, and touch interfaces in particular, that allow senior users to have an improved user experience. Moreover, following the conclusion from previous literature [3, 8, 12, 17, 18], one might consider that it would be interesting to have a mobile social network, which could make use of its communication technologies [8] in order to bring family proximity [14] to the senior users, while providing a better user-experience based on its interface characteristics [20].

## 3. Characterizing Seniors' Usage of Facebook

With the goal of understanding and characterizing older adults usage of social networks, we started our work by conducting a series of user studies, following a mixed methods approach, combining focus groups, interviews, and direct user observation. The methods used in our work are based on an extensive literature revision that has given us several supportive indicators that show that these approaches result in a better perception of the user's interaction with SNS, and concretely the Facebook platform. Our preference for these approaches was recently justified by Langdon et al. [22] as their approach has considered the advantages of triangulation of data sources [23]. Following this method, data is collected from multiple approaches such as literature studies, quantitative data analysis of data from user trials, user surveys, focus groups, and qualitative analysis of observational data from user interviews [23]. Recently, Coelho et al. [24] also used such methods when they have managed to collect end user requirements for older adults resorting to a mixed methods approach.

In the first phase, we conducted interviews and focus groups with a total of 20 participants, in two distinct institutions: senior healthcare institution and senior university. In a second phase of the study, we ran an experimental session with two older adults (aged 86 and 62 years old) interacting with Facebook on a tablet device. With the results from those studies, we compiled a set of design recommendations for the design of social network interfaces with a focus on senior users.

Privacy was the most discussed issue with the participants and the main reason appointed by seniors for not using Facebook, or using it in a limited way. Some participants were even reluctant to see information related with people they did not know, and thus, they suggested the *modification of some Facebook's privacy options: “Post to” and “View from,”* making them configurable. The second issue discussed was having *group-based content and functionality*. Recommendations suggested the inclusion of a “favourites” group with the most interacted with contacts, to and from whom the user could post and see updates. Although Facebook already creates a predefined group called “favourites,” in this group users have to manually define who are their favourite contacts. *Special relevance was given to the family group and family-based functionalities*, as a step for more isolated seniors to adopt Facebook. Participants gave several indications that interaction should be focused in family, which is supported by the fact that several participants reacted positively every time they saw information related with a family member and by writing family-related posts. Moreover, some participants also suggested the *inclusion of family-based features, such as the capability of creating family events*. Related with Facebook content, participants have shown different reactions to different types of content, and thus recommendations are to give *more relevance to photos and images than to other types of content*. Further, participants have suggested that the Facebook newsfeed should include some mechanism for media content filtering purposes. Also, participants suggested that *functionalities related with “friends of friends” or “getting to know new people” must be avoided or made optional*. Regarding interface concepts, participants suggested that it must be simple, constant (since the Facebook interface is updated too often), and *provide “safe points,” that is, clear ways for the user to return to the homepage of the application*, as well as avoiding nonnative language interface terms and content.

## 4. A Facebook Interface for Seniors

**Figure 1:**
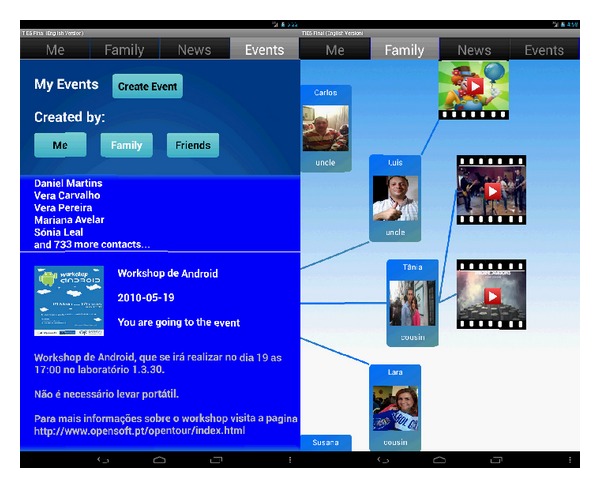
Prototype's support for *event management* (left) and the *family graph* presentation (right).

The aforementioned recommendations and requirements were considered when prototyping a tablet interface for seniors accessing Facebook services. The prototype (Figure 1) has three main focus areas: *content publishing, content visualization*, and *events management*.

The *content publishing* functionality addresses the privacy concerns. The prototype provides a mechanism that allows the user to choose to whom his or her post will be visible: family, friends, or friends of friends. This feature is easily accessible while posting any update. Although this feature is already implemented in Facebook Mobile, several of the participants in the aforementioned studies did not know about its existence, which indicates its usability can be improved.

The recommendations regarding the *content visualization* lead us to focus on the prototype in family-related contents. We developed a family graph presentation (Figure 1, right) that renders the user's relatives within a circular structured graph. We believe that the chosen graph-based approach suits the needs of the senior population regarding family-based content presentation and visualization, by clearly presenting the relations between family members. Moreover, we implemented a mechanism to present the media contents that were posted by each of the user's relatives, as well as a mechanism that allows the user to filter by content type, or by the number of likes.

Facebook already provides filtered access to all the information that is related to a specific list within the user's social graph, through friend lists automatically created and prefilled based on users and their contacts information. In our prototype we implemented a newsfeed visualization functionality, providing the user with the ability to filter the contents that were either posted by the user's family, friends, or him/herself. *Note that this solution differs from the aforementioned graph-based one, since it provides the user with the ability to visualize the information in a different way, closer to what Facebook already employs*.

Additionally, we have implemented *event management* functionalities, supporting event creation, edition, and deletion. Functionalities also include a filter mechanism to allow the user to see the events that were created by him or herself, friends, or friends of friends. Further, this functionality also allows the user to edit an event that was created by him or her, giving support for inviting additional contacts, or uninviting contacts already invited.

## 5. Comparing the Prototype with Facebook's Mobile Application 

### 5.1. Procedure

To assess the effect of the design recommendations, we conducted a user study where we compared Facebook's Android native application and the prototype designed according to the design recommendations.

The study was conducted both in a senior nursing home and a senior university, with 10 different participants. Every participant already had a Facebook account, but they had never used Facebook's native application on a mobile device. The average age of the participants was 72 years old, with the oldest participant being 81, and the youngest being 67 years old. Only two participants were not frequent users of SNS. Seven out of ten participants stated they used the SNS to see news about their friends and family. Eight out of ten participants stated he or she feels that SNS provide a feeling of closeness to his or her family, mainly by providing easy means of communication. Quoting some participants: “[*⋯*] if Facebook lets me talk to my sons, then I feel close to them!”, “Sure! It is easy and cheap!”.

We started by presenting our work and goals of the study. Participants were then asked to perform 9 tasks in both Facebook's native application and our prototype. The order of presentation of the two applications was alternated, with half of the participants performing the tasks first in Facebook's native application and the other half in our prototype, in order to avoid bias of participants' reactions and responses.

To make sure the selected tasks were representative of real usage of Facebook's services, we asked the participants, for each task except the first one (Login), to describe a real usage scenario in which they would use the feature the task was requesting them to use. Overall, participants' responses were uniform. They often want to share messages to only a selected group within their contacts (*second task*). Participants also stated that they use the SNS to see news updates, especially regarding their relatives, as well as their profiles (*third, fourth, and fifth tasks*). One of the reasons that participants used to justify their tendency to want to see their family in the SNS is the fact that they usually love to see photos and interesting videos they have posted (*sixth task*). Lastly, it was curious to see that every participant was in accordance when answering the question “What would you like to say to your family?”, since all responses showed that participants are often worried about inviting their relatives to events they want to organize, but do not know how to broadcast the message (*seventh, eighth, and ninth tasks*).

After each task, participants were asked to qualify the task according to both its difficulty and usefulness, on a scale from 0 (lowest) to 5 (highest). We also recorded participants' comments and reactions and performed a qualitative analysis, of which we present our findings in the next section.

### 5.2. Results

Table 1 presents an overview of the participant's perceived task easiness, in both native application and prototype, and usefulness. As can be seen, tasks in the prototype were perceived to be easier to perform. With the exception of reading the newsfeed, all other tasks were notoriously easier to perform with the prototype. This is particularly relevant, given that tasks were focused on the features that seniors attribute bigger importance for using a social network (according to what was collected in the initial studies). Strengthening this is the fact that all tasks, and therefore, all functionalities associated to them, were considered useful. In the following section we present a quantitative and qualitative overview of these results, from the trial's observation and participants' comments.

### 5.3. Analysis

#### 5.3.1. Quantitative Analysis

**Figure 2:**
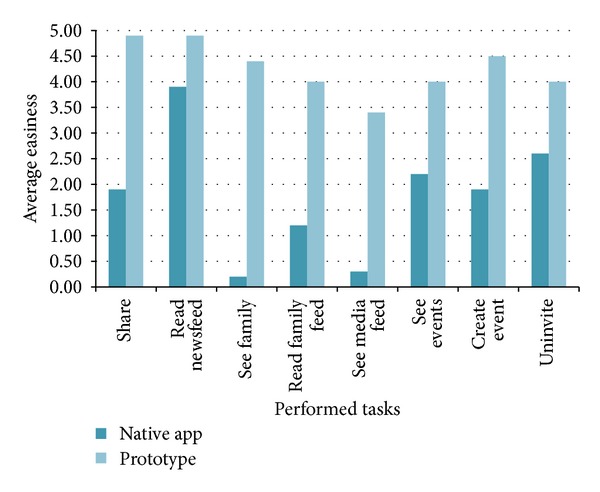
The graph of participants' perceived *usability* regarding the user studies' tasks.

Figure 2 illustrates the discrepancy of quantitative results, regarding the tasks performed in both approaches (the first task—Login—is not presented, since we did not collect any quantitative measures for it). The graph represents the average of results presented previously in Table 1, in which the values are accounted from 0 (very hard) to 5 (very easy). From such illustration we can see that when asked to *share* something to their contacts, participants have felt greater easiness performing the task in our prototype (4.9 out of 5 points, average), when contrasting with the Native Application (1.9 out of 5 points, average).

**Figure 3:**
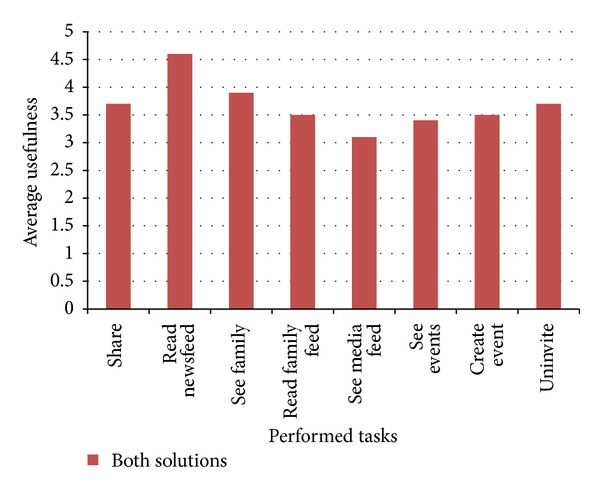
The graph of participants' perceived *usefulness* regarding the user studies' tasks.

Further, *reading the newsfeed* was considered an easy task to perform in both approaches, by the participants. Even so, participants have felt greater ease of interaction when performing the task in the prototype (again, 4.9 out of 5 points, average), rather than when performing the task in the Native Application (3.9 out of 5 points, average). This value closeness—contrastingly to the previous results—can be justified by the fact that the participants are first presented with their newsfeed, when accessing their account via the Native Facebook Mobile Application. Moreover, participants have considered this task as one of the most important for them, as suggested by Figure 3, with the task obtaining 4.6 out of 5 points (average).

The task in which the participants have reported more difficulties performing in the Native Application was the fourth, that asked them to *see their family*. As shown in Figure 2, we can observe that there was a huge difference between the easiness of performing the task in the two applications. There was a clearer ease of interaction being reported by participants when performing the task in the prototype (4.4 out of 5 points, average), contrasting with the difficulties reported in the Native Facebook Mobile Application (0.2 out of 5 points, average).

When asked to *read the family feed*, participants have reported a greater ease of interaction performing the task on the Native Application (1.2 out of 5 points, average), comparing to the previous task. Still, this result is clearly representative of the challenges that the participants have felt when interacting with the Native Application, and this becomes more notorious when comparing the numeric results with the ones obtained in the prototype (4 out of 5 points, average). Also, according to our previously mentioned research, we have obtained quantitative results that show that these family features are regarded as important for the older adults, as has been suggested (Figure 3).

Moreover, *seeing the media feed* was also a task in which the participants have felt great difficulties, when interacting with the Native Application (0.3 out of 5 points, average). Further, this task has been easily performed in the prototype by the participants (3.4 out of 5 points, average). This value is a strong indicator that the Native Facebook Mobile Application has no usable support to filter the media content that is presented in the users' newsfeed.

Further, *seeing events, creating events*, and *uninviting contacts from events* were tasks in which the participants have also reported a greater ease of interaction in the prototype, with this approach obtaining about 2 more points (out of 5, average) in each of the aforementioned tasks. Also, Figure 3 clearly shows that the participants have considered this type of functionalities very useful, with the three tasks that comprehended event management activities having around 3.5 points out of 5 (average).

#### 5.3.2. Qualitative Analysis

For a qualitative analysis we will divide the results into five distinct groups. The groups are directly related to the way we grouped the study tasks: *posting tasks, find contacts that shared a kinship relation with the user, content presentation, content filtering*, and *event management*.

Results from the user studies have shown good support for us to consider that the Native Facebook Application requires some interface improvements, considering the design recommendations in which we focused in. Participants' initial reaction to Facebook's Native Application interface revealed some confusion. This was due in part to the large amount of information that was presented in the interface—note that the first screen presented in the native application is the newsfeed. Some of the participants' reactions were “What is this? This looks different from the computer version!” or “I do not even understand this! I will not touch it!”. Contrastingly, participants' reaction to our Facebook-based prototype revealed enthusiasm for the contents presented (their profile), as commentaries show: “Oh look, it is me, so cute!” and “This is so beautiful! How did you do it?”.

When faced with the *posting tasks*, participants revealed a much greater efficiency when performing them on our prototype. When using the Native Application's interface, not a single participant could easily find the right way to post a message on his or her wall. In addition, after finding out how to post a message, participants were not able to understand how to configure the privacy of the post itself. Some participants' most relevant commentaries supported those facts: “Where do I find it?” or “Where do I change the target audience of the post?! I cannot find it.”

We obtained similar results regarding tasks in which the participants were asked to *find contacts that shared a kinship relation with them*. Participants revealed great difficulty when searching for their relatives within the native Application. This was due to the fact that in order to see his or her family, participants had to first click the top-left corner button, which, as some participants stated, gives no clue about its functions. After clicking the button, participants had to cycle through a big list, in order to finally see their family. As a result, only two participants found it not hard to perform the task in the native application, with the remaining participants considering the task hard to perform. Some participants even considered the task “impossible” to perform “if not assisted” by the user studies moderator, as some of their statements have shown. For the prototype every participant acknowledged the task as being very easy to perform.

Considering tasks related to *content presentation*, after the difficult task of discovering how to see their family, participants found it easier to browse the contents shared by their kin in the native application. This can be expected, since that is the content presented after selecting the family group in the options menu. Likewise, on the prototype, participants found it easy to search for their family's activity, since they only needed to select the “News” tab, which opens up by default to the family newsfeed. Participants stated the task was “very simple to perform.”

When considering *content filtering*, every participant found difficulties when interacting with the native application. This was due to the fact that the native application does not possess any media content filtering mechanism, which makes the task “very hard to perform,” as some participants stated. Likewise, on the prototype, participants have revealed some difficulties when performing the aforementioned task. Some of them stated that since they were already presented with their newsfeed they expected it to provide some filtering mechanism in order to easily find different media content. Nonetheless, after finding out that the media content filtering mechanism was embedded in the family graph, participants considered the task very easy to perform, mainly due to the “information disposition” and “ease of interaction.”

Regarding *event management* activities, participants felt great difficulties when interacting with the native application. This is explained by the fact that the process to find user's events within the application is analogous to the one required to find the participant's family: the participant had to click the top-left corner button and then wander through a big list, such that he or she could finally see his or her events. In addition to this, participants were not able to easily find out which events had been created either by them or their contacts, since (as some of them stated) the information was “too confusing.” Moreover, creating an event revealed to be a complicated task for some participants, when using the native application. This results from the “create event” button being represented as a “+” signal, which gave no notion of its main functionality to our participants. Contrastingly, on the prototype, participants revealed great ease of interaction with the interface. They were able to freely see all of the events from different sources, as well as create events of their own. Some of them also stated that the event management was “very easy,” since the buttons are “very well labelled.” Additionally, participants were easily able to uninvite contacts from their own events.

### 5.4. Discussion

We conducted a user study, comparing the current native Facebook application for mobile devices with our prototype. Results from the user study show that senior users experience several difficulties when performing family related tasks in Facebook's native application. On the other hand, with our prototype designed according to the elicited recommendations, they were able to easily complete most of the requested tasks, revealing high levels of satisfaction. Consequently, we have preliminary indications that our design recommendations do contribute to improve the usability and accessibility of social networks, which can lead to improving seniors' quality of life.

Results from our user studies have shown that there are several issues to be considered in order to improve the native Facebook application, to make it more usable by the senior population. Participants' initial reaction to Facebook's Native Application interface revealed some confusion. This was due in part to the large amount of information that was presented in the interface—note that the first screen presented in the native application is the newsfeed.

Our quantitative analysis has shown that tasks in the prototype were much easier to perform, with subjective easiness measures revealing a great discrepancy when tasks were performed in the prototype, in contrast with tasks performed in the Native Facebook Mobile Application. With the exception of reading the newsfeed, all other tasks were notoriously easier to perform with the prototype. Another indicator of this is the fact that all tasks, and therefore, all functionalities associated to them, were considered useful.

Moreover, our qualitative analysis to the results obtained in the studies has also shown that the tasks were much easier to perform in the prototype. This has been mainly supported by the participants' quotes and reactions when interacting with the Native Application: “Where do I find it?”, “Where do I change the target audience of the post?! I cannot find it”—when trying to post something in his or her wall; “This is impossible!”—when trying to find contacts that had a kinship relation with him or her; “Without help, I will not be able to do it!”—when trying to find and filter media content presented in his or her newsfeed.

Summarising, there was a straight concordance between qualitative and quantitative results. Every task to which the quantitative results pointed as being easy to perform in the solutions has also been quoted by the same participants as being “easy to perform.” Moreover, the metrics that were applied to measure the task's usefulness demonstrate that the functionalities and the focus that was given to the prototype is the correct one, for this set of user population.

Thus, we have considered as main findings and outcomes of the user study performed in this phase of our work, that a prototype that was designed under the recommendations derived previously in our work is more adequate to the elderly population than the Native Facebook Mobile Application. Some crucial aspects provided by the prototype include facilitating the interaction and potentiating activities that allow users to post messages to a directed and determined group of users. Also, this type of action was made available in order to provide the same mechanism for older adults to easily post content to their family, assuring that the user is granted the required privacy. Moreover, there was large focus onto family activities, such as granting the access of a user to his or her family.

The family presentation was also an interesting point in which we brought insight, since results have shown that participants of our study feel great affinity by the type of interaction provided by the graph structure that was used in our prototype solution. Therefore, we suggest the inclusion of this type of structure in the Native Facebook Mobile Application, providing the older adults with a clearer view over their family members, as well as a greater user experience when navigating their genealogical tree.

Also, given our study results, we have strong indicators that there should be additional support for the promotion of the users' family posted media content. This becomes crucial if we take into account that the Native Facebook Mobile Application has no real support for content filtering. Thus, without such mechanism, there is a clear challenge for older adults—and users in general—that might probably want to visualize their contacts' posted media content.

Lastly, our results have pointed out that event management activities should also be fostered, since as our user studies' participants have stated, events “allow the grouping of family members” and therefore “support closeness to family.” This is also a critical issue that has been considered and implemented in our prototype solution. Contrastingly, the Native Facebook Mobile Application has not a usable support for event management. Moreover, the Native Facebook Mobile Application also does not provide the users with the options to invite a specific group of contacts to their newly created events. This represents a challenge for older adults who have reported they would mainly use the event management mechanisms in order to invite their relatives to family events.

## 6. Conclusions

Social networks have the potential to mitigate social isolation problems felt by seniors worldwide. However, currently, the social networks which support the largest number of users do not cater for the requirements of older adults. We have developed several elicitation activities that allowed us to identify a set of design requirements for social networks that aim to address these concerns. We found that privacy and a big focus on family related content and activities are paramount for senior users.

Our research revealed that the current Native Facebook Mobile Application is not immediately usable by older adults. In many cases, they do not have a clue of where (in the application) they can perform their desired activities, and therefore demonstrate several difficulties performing those activities. Thus, we have discovered that the range of actions that older adults often want to perform in the scope of a Mobile SNS can be mapped into several grouped behaviours. Those behaviours were then derived into the form of recommendations for the design of a Mobile SNS. Accordingly, we managed to develop a Facebook-based prototype for tablets that focuses on the older adults' desired behaviours. Further, we used the developed prototype to compare its usability (older adults' level of satisfaction, concretely easiness, and usefulness) against the Native Facebook Mobile Application. We have then managed to unveil that the prototype provides the older adults with high levels of easiness of interaction—regarding the tasks that they often want to perform in Mobile SNS—contrasting with the Native Facebook Mobile Application, in which the older adults feel serious difficulties performing their desired behaviours, mainly due to the interface limitations.

However, some issues in our approaches can still pose limitations to the sharpness of the results. As future work, it would be interesting to compare the older adults' usability with a larger number of participants. Also, we plan to conduct our future studies with a more broader scope of context, comparing the older adults' interaction not only between our Facebook-based prototype and the Native Facebook Mobile Application, but also with the Desktop version of the Facebook SNS. Finally, we plan to make use of more specific and accurate measures of usability—including efficiency and effectiveness—and also perform a more complete study—for example, diary studies of older adults' interaction with the aforementioned applications—in order to obtain several indicators of how older adults could benefit from SNS in general, to improve their quality of life. Moreover, we intend to understand which of the results that were obtained from our work are also applicable for other SNS, such as Twitter or Google+. By doing so, we aim to perceive if such SNS have different characteristics, as well as get to know if a novel type of mechanism could be or should be used, in order to make them more usable and accessible for older adults.

## Figures and Tables

**Table 1:** Average subjective easiness in both the native application and prototype, and perceived usefulness for each task. Scale range from 0 (lowest) to 5 (highest).

Number	Task description	Easiness		Usefulness
		Native App	Prototype	
1	Login	—	—	—
2	Post	1.9	4.9	3.7
3	See newsfeed	3.9	4.9	4.6
4	See family	0.2	4.4	3.9
5	See family feed	1.2	4.0	3.5
6	See media feed	0.3	3.4	3.1
7	See events	2.2	4	3.4
8	Create event	1.9	4.5	3.5
9	Uninvite	2.6	4	3.7
